# Exploring experiences of pharmacists and pharmacy students using mHealth apps: A qualitative study of user experience

**DOI:** 10.1080/17482631.2023.2245606

**Published:** 2023-08-10

**Authors:** Jovana Ilkic, Andrijana Milosevic Georgiev, Dragana Lakic, Valentina Marinkovic

**Affiliations:** a Student at Faculty of Pharmacy, University of Belgrade, Belgrade, Serbia; b Department of Social Pharmacy and Pharmaceutical Legislation, Faculty of Pharmacy, University of Belgrade, Belgrade, Serbia; c Teaching Assistant with PhD at Faculty of Pharmacy, University of Belgrade, Belgrade, Serbia; d At Faculty of Pharmacy, University of Belgrade, Belgrade, Serbia; e Full Professor and Head of the Department at Faculty of Pharmacy, University of Belgrade, Belgrade, Serbia

**Keywords:** Digital health, person-centred care, pharmaceutical care, UX research, mobile technology

## Abstract

**Purpose:**

Pharmacists can enhance mHealth delivery by promoting the awareness and use of health apps, while pharmacy students and early career pharmacists allow broader perspective for further development. This study aimed to explore the experiences and attitudes of pharmacy students and pharmacists, on the use, advantages and possible improvement of available mHealth platforms in Serbia.

**Methods:**

One-on-one interviews were conducted online in two phases, during March and April 2020 (*n* = 40) and during March 2021 (*n* = 20), using a published, validated interview guide which was adapted for this study. Interviews were transcribed ad verbatim, coded and thematically analysed.

**Results:**

Although participants recognized room for optimization, most apps had satisfactory user experience. Participants used health apps as a source of updated information and tools in education and work, but also for self-development. During working hours, participants mostly used medication-related apps, however, it was shown that they use different health apps in order to provide the best possible care to patients.

**Conclusions:**

Greater insight into usage, expectations, benefits and challenges of mHealth in pharmacy was obtained and can lead into better informed design of health apps to encourage students and pharmacists, both as professionals and individuals, on the path of their continuous improvement.

## Introduction

In the world of continuous technological development, there is a constant demand for the improvement of healthcare systems and services, primarily through the adoption of innovation to provide high quality personalized care adapted to the needs of patients (Gregório et al., 2021). Healthcare digitalization has the potential to improve quality and equity when it comes to health and well-being of society members, as well as to support the increase in their independence and involvement (Cuesta et al., 2020). As a branch of digital health (Smith & Magnani, 2019), mHealth represents the use of mobile technologies, with an emphasis on mobile applications, in order to achieve improved health outcomes and quality of life, both through self-care (Anderson et al., 2016; Obro et al., 2021) and by helping healthcare professionals in providing efficient and adequate care to patients (International Pharmaceutical Federation [homepage on the Internet], 2022). Critical aspects of person-centred pharmaceutical care include listening to an individual in order to gain a better understanding of their needs and perspective, as well as providing information in a way that enables the person to make informed decisions and supporting them to develop goals related to their lifestyle, health and treatment (Marinkovic et al., 2022).

Mobile apps can help individuals manage diseases or conditions, improve medication adherence and facilitate medical history recording. On the other hand, using such apps aids pharmacists in keeping up with disease management guidelines, maintaining adequate pharmacy supplies, accessing drug information systems and patient health information, as well as using tools to calculate drug doses and accurately convert between units of measurement (International Pharmaceutical Federation [homepage on the Internet], 2022). mHealth contributes to maintaining and improving patients’ health, safety, and quality of life while providing meaningful real-time data to patients and healthcare professionals, emphasizing on user experience (UX) design. Optimizing the UX design during app utilization, increases the app’s usability, usefulness and attractiveness, and most importantly, user satisfaction, which leads to users actually using the available applications. It also leads to a better understanding of user needs, which in the case of mHealth could mean patients, that is, users of health services (Kirkscey, 2021). Although mHealth is making steady progress in global healthcare, data privacy risks, variable information literacy, and Internet access requirements are barriers between patients/healthcare providers and the ability to take full advantage of mHealth technologies (International Pharmaceutical Federation [homepage on the Internet], 2022). Additionally, patients who use information technologies tend to know more about their conditions and want to participate in healthcare decision-making, but doctors may consider them “difficult [and/or] demanding” (Kopelson, 2009). However, that is precisely why further user experience research of all interested parties is necessary for the optimization to be successful (Kirkscey, 2021).

In addition, mHealth displays a promising impact on healthcare delivery in low- and middle- income countries (Hurt et al., 2016), such as the Republic of Serbia (Serbia) (Low & middle income [Internet]. The World Bank, 2022), while pharmacists have a good placement in health systems to promote the use of health apps and make patients more aware of them (Kayyali et al., 2017). Perceptions of pharmacy students and early career pharmacists provide an important perspective that complements the picture held by more experienced pharmacists (Davies et al., 2015; Meilianti et al., 2022). Therefore, this study was designed to explore the experiences and attitudes of pharmacists and pharmacy students from Serbia on the use of available apps related to the use of medicines, cosmetic products and healthy lifestyles, with the aim of evaluating the way of use, advantages and room for improvement when it comes to available mHealth platforms and tools.

## Materials and methods

A qualitative study of the user experience of pharmacists and pharmacy students using health-related mobile apps was conducted online via structured one-on-one interviews. The interviews were conducted in two phases. During March and April 2020, pharmacy students and masters of pharmacy who graduated recently (up to 2 years after obtaining their degree of Masters of Pharmacy) were interviewed. Afterwards, in March 2021, interviews were conducted with pharmacists with more than five years of work experience. The research was approved by the Ethics Committee of the Pharmaceutical Chamber of Serbia (approval number: 262/1–2).

### Interview design

Interviews were conducted anonymously and voluntarily. Before the interview, each participant was informed about the study concept and interview questions, as well as the confidentiality and storage of their data. Subsequently, the participants were asked to provide their informed consents.

The questions used during the interview were taken from the paper *“Mobile Health Apps to Facilitate Self-Care: A Qualitative Study of User Experiences”* by Anderson and colleagues (Anderson et al., 2016), with the original paper’s authors’ consent. The interview guide in the original paper was based on several of the most relevant theoretical frameworks for UX research—Technology Acceptance Model (TAM), Health Information Technology Acceptance Model (HITAM) and Mobile Application Rating Scale (MARS), and was adapted to a holistic exploration of various health-related mobile apps use (Anderson et al., 2016).

TAM quantifies the acceptability of technology from the user’s perspective, and its main determinants are the perceived usefulness and perceived ease of use of the researched technology (Davis, 1989). However, health behaviour is too complex to be described by a single model or theory. Consequently, by implementing health behaviour theories within the application of health information technology (HIT), the applicability of TAM was expanded, and HITAM was created. This model provided data on personal and social factors such as motivation to use the application, self-reflection, competition with other users and the user’s desire to further recommend the application (Anderson et al., 2016; Kim & Park, 2012). MARS is a simple, objective and reliable tool for classifying and evaluating the quality of mobile health applications. This scale includes four quality factors, namely functionality, engagement, aesthetics and the quality of the information provided, which were also incorporated into the interview. Concepts that intertwine between these models have been eliminated (Anderson et al., 2016; Stoyanov et al., 2015).

Questions were translated into Serbian, considering that the research was conducted in Serbia and that it would be significantly easier for the respondents to be questioned and give answers in their native language. The questions were translated directly from English to Serbian by one of the researchers. Then another researcher translated the questions from Serbian back to English to ensure that the essence of the questions was preserved (Abfalter et al., 2020). After that, the researchers discussed both translations and agreed on a set of questions to be used during the research, with the addition of two demographic questions, to gain a better insight into the study population (gender and level of education of the respondents).

The interview guide consisted of 24 main questions, beyond which supplementary questions were raised in case there was a need for further clarification. The first five questions were used to collect participants’ demographic data (age, gender, occupation, years of work experience, level of education), while the other 19 questions were related to mHealth apps and experience of the users. The participants were asked which health apps they used and if they could elaborate on how they used one of them. Consequentially they were asked how they discovered the app, about the user profile set up and for how long they used it, what they liked about it and how easy to use the app was, whether they didn’t know what to do with the app at any point and whether they noticed any bugs or shortcomings. In addition, there were queries about data storage, notifications, conflicting features, app performance speed, the need for peripheral devices, what users regarded as advantages and disadvantages of the app(s), whether they faced any technology or terminology they weren’t familiar with, as well as what alterations they would like to see. The researchers conducting the interviews raised the questions in the same order, and each interview was planned to last about 15 minutes.

### Sampling and recruiting

Students, early career pharmacists (Masters of Pharmacy who obtained their diploma in the past two years) and more experienced pharmacists (pharmacists with more than five years of work experience) from different cities of the Republic of Serbia were included in the research using the snowball method (Sheu et al., 2009).

Four sets of criteria were established for the inclusion of respondents, as listed below:

1st Set of criteria: A candidate is a pharmacy student or a pharmacy graduate who obtained their degree in the previous two years, and who uses or has used an application related to the use of drugs or cosmetic products.

2nd Set of criteria: A candidate is a pharmacy student or a pharmacy graduate who obtained their degree in the previous two years, and uses or has used apps related to healthy lifestyles (nutrition, exercising, etc.).

3rd Set of criteria: A candidate is a licenced pharmacist who works or has worked in a pharmacy, with more than five years of work experience and applies or has used applications related to the use of drugs or cosmetic products.

4th Set of criteria: A candidate is a licenced pharmacist who works or has worked in a pharmacy, with more than five years of work experience and uses or has used applications related to healthy lifestyles.

Finally, the inclusion criteria applicable for all participants were that they were of legal age, had access to the Internet, had knowledge of digital technologies, and that they were familiar with Cisco Webex Meeting software.

When no new codes and themes emerged during the interviews, it was agreed that data saturation had been reached and that there was no need for further interviewing (Guest et al., 2006). Based on the inclusion criteria, the respondents were grouped into three clusters—the first cluster (Cluster I) corresponding to the 1st set of criteria and the second cluster (Cluster II) to the 2nd set of criteria. The third cluster (Cluster III) included the 3rd and 4th set of criteria, as only a few pharmacists wanted to discuss applications related to healthy lifestyles, while new codes quickly stopped being generated.

Setup, data collection and data management

The interviews were conducted online using Cisco WebEx video conferencing platform. These were carried out outside of participants’ working hours or classes, when participants were at their homes, where they arranged to be alone in the room and in a quiet environment. Only the researcher and a participant were present during the interview to ensure there were no distractions during the meeting. Each interview was recorded and stored in researchers’ WebEx recording storage locations. No one except the researchers who conducted the interviews had access to this data. Each interview was transcribed verbatim, and the answers were coded and analysed.

### Data analysis

For data analysis, the six-step thematic analysis approach developed by Braun and Clarke was applied (Braun & Clarke, 2006). To begin with, (1) the recorded material was continuously listened to, while the researcher took notes on the initial analytical observations. Once the recorded materials were anonymously transcribed, this was followed by: (2) Generating initial codes, which grouped the data into meaningful units, by combining inductive and deductive coding (inductive codes were derived from the interviews, while deductive codes emerged from the themes used in the interview guide); After thoroughly reviewing the recordings, understanding the scope and context, and organizing data into themes, groups of codes were extracted with the aim of mapping key patterns; (3) By grouping the codes, broader themes emerged; (4) Team members further analysed the data, compared the selected topics and resolved disagreements through discussion until reaching an agreement on the topics; (5) Final definition and naming of topics; (6) Data analysis and reporting (Braun & Clarke, 2006).

Results were analysed in Serbian. The main themes and subthemes were generated, and the participants’ quotes were translated into English to present the results in the international literature. The translation was done by the research team, using informal language to reflect the actual type of language used during the discussion.

## Results

### Characteristics of participants and mHealth apps

60 respondents who participated in the research were grouped into three clusters equal in size: Cluster I (Participants P1-P20), Cluster II (Participants P21-P40) and Cluster III (Participants P41-P60). Clusters I and II consisted of pharmacy students predominantly and a few Masters of Pharmacy, whilst Cluster III included 20 pharmacists with more than five years of work experience, with a half being Pharmacy Masters and the others being Pharmacy Specialists. Within Clusters I and II, most respondents were between 18 and 25 years old, without any work experience, unlike Cluster III, while in all clusters, there were more female respondents. More detailed demographic data for all three clusters are shown in Table I.

**Table I.** Demographic data of the participants.

		Cluster I (n*)	Cluster II (n*)	Cluster III (n*)
Gender	Male	5	1	5
	Female	15	19	15
Age	18–25	19	20	-
	26–35	1	-	4
	36–45	-	-	11
	46–55	-	-	3
	>55	-	-	2
Education	Pharmacy Student	17	17	-
	Masters of Pharmacy	3	3	10
	Pharmacy Specialist	-	-	10
Work experience (years)	0	18	19	-
	1–2	2	1	-
	5–10	-	-	10
	11–20	-	-	5
	>20	-	-	5

*Note*: *The results in Table I are displayed as number of participants (n).

The interviews lasted approximately 11 minutes on average across all three clusters (the longest interview lasted 25 minutes, while the shortest one was finished in 5 minutes), during which most interviewees managed to answer the key questions in accordance with their experience. Out of 60 participants, 7 participants didn’t answer 1 question, while 1 participant didn’t answer 2 questions during the interview. This happened due to interruptions (e.g., someone entering the room the participant was in during the interview, participant’s phone ringing) or participants not being sure how to respond to a question.

The participants mostly reported using or having used recently (up to 6 months before the interview) apps that provide information about medications (7 types), skin care (1 app), and fitness apps (11 types). However, they also mentioned using apps for running and/or walking (4 types), weight and calory management (3 types), water intake (1 type), menstrual cycle monitoring (1 type), diabetes management (1 type) and pregnancy monitoring (1 type). Table II displays the app type participants decided to discuss further during the interview. Participants within Clusters I and II mostly used the mentioned applications for less than 2 years, while respondents from Cluster III mainly used these apps longer than 3 years. Cluster I participants mostly discovered apps through the recommendation of colleagues. A few heard about apps from family members, friends, on social media, at college (medication apps), or during skin care product advertising (skin care apps). Within Cluster II, respondents massively discovered apps through independent research, or by getting them with the phone or through a friend’s recommendation. A few heard about the app/s on social media. Cluster III participants learned about these apps independently or through the recommendation of colleagues, the Chamber of Pharmacy or business partners. Two respondents from Cluster III did not remember how they discovered the app because it had been a long time since they started using it. The majority of respondents (from all Clusters) used Android, while the rest used iOS operating systems.

**Table II.** mHealth app types which were discussed by the participants during the interviews.

App type	Participant’s code	Total
Skin care	P1, P2, P3, P4, P5, P6, P7, P8, P9, P10, P57, P60	12
Medication use/information	P11, P12, P13, P14, P15, P16, P17, P18, P19, P20, P41, P42, P43, P44, P45, P47, P48, P50, P51, P52, P53, P54, P55, P56, P59	25
Fitness	P21, P22, P23, P26, P29, P30, P31, P33, P34, P35, P36, P37, P38, P40, P46	15
Running and/or walking	P24, P27, P32, P39, P49	5
Weight and calory management	P25, P28	2
Pregnancy monitoring	P58	1

User experience—Thematic analysis

Based on the thematic analysis, several key themes were identified—usability and functionality, engagement, information, as well as perceived usefulness and value for the user. The final mapping of themes and sub-themes is presented in Table III.

**Table III.** Final thematic framework including main themes and sub-themes.

Theme	Sub-theme
Usability and functionality	Efficiency
	Detected app problems
	Feedback
	Aesthetics
Information	Information quality
	Data privacy and storage
Engagement	Interactions
	Motivation and satisfaction
Perceived usefulness and value for the user	Professional and/or educational value
	Personal value
	Expansion

### Usability and functionality

#### Efficiency

Participants from all clusters mostly believed that their apps were easy to use and could quickly perform desired actions without using multiple apps in parallel. Especially in the case of apps used during working hours because they provide essential information and news about medications and their use, it was emphasized how important it is that the application is easily accessible, consistent, practical and time-effective.

The app is always on my home screen, promptly available when I need to read some news or check something I need in a short period of time in the pharmacy regarding drug parallels, the method of [drug] application, [drug] interactions, contraindications, dosage method so, more for consolidating already existing knowledge and if possible, for following news… (P41)

The only respondent who felt that the app was not that easy to use, talked about a fitness app that had an option to track calories consumed, as there was no easy way to calculate the value of a portion consumed, within the app.

#### Detected app problems

Although the participants emphasized that there is a meaningful sequence of activities within the apps and that they are easy to use, they mentioned that they faced issues while using them. The most frequently highlighted challenges were bugs, the need to reinstall the app to unfreeze it, problems when logging in, i.e., failure to recognize the user account, impossible or slow updating, problems with synchronization with peripheral devices (watch), tedious registration process, lack of data synchronization, overly complicated and unfamiliar content, or application content not available in the local language. Most of the bugs were reported by users who talked about the apps related to skin care, while there were very few or no bugs when it came to the other mentioned applications.

When I use the app continuously for 6 days, it crashes on the seventh day. That is, it [the app] keeps saying an error has occurred, … I can’t do anything, I have to literally uninstall it, reinstall it, log in, and then I can use it. I mean, I told that to the representatives [of the company whose application is], but I thought that I did something wrong. (P60)

Additionally, respondents who talked about apps related to medications or their use (Cluster I and Cluster III) cited more challenges at the content level—complexity, unfamiliar tools and the fact that some apps/tools do not exist in the Serbian language. Although these challenges were mentioned within both clusters, younger participants (Cluster I) emphasized these challenges more often than older ones (Cluster III).

[Within the app] There are some tools that are not explained in detail what they are for. It might be convenient if more information about these were available. (P14)

There was some medical terminology related to the names of some tests, but as the application is intended for other health professionals too, I did not get overly excited because it is not something I would use in pharmaceutical practice, but of course out of curiosity I looked it up. Not in the sense that it was unclear, but just really unknown, I’ve simply never heard of that terminology and the names of the tests that are done for some of the parameters. (P42)

#### Feedback

Participants acknowledged the presence of visual stimulations (notifications) from the app significantly more than the audible ones, with many respondents explaining that they keep either the app(s) or the phone on silent mode as they do not prefer that type of notification. The importance of sound notifications was highlighted by fitness and walking/running apps, as a signal that a certain goal has been reached (mileage covered, exercise done) or as a reminder to be active.

When I’m moving, when I cross a kilometre, I hear the application saying you’ve crossed the first kilometre and it says some basic information, e.g., at what speed I am moving and it repeats all that for every following kilometre. (P32)

The majority of respondents stated that they prefer visual notifications over tactile (vibration), with a few stating that they have no preference.

#### Aesthetics

Most respondents had no objections to the aesthetic aspect of the applications they used. They emphasized that they were satisfied with simple design, the display of information and adequate font. Only the skin care app users pointed out the lack of visibility of certain segments.

On a scale from 1 to 10, let [the appearance of the app] be 9. I really like the appearance of the application, it is very reduced, the colour is greyish, the size of the letters is suitable, however in the description of some products the letters are smaller, which is sometimes a problem for me because I wear glasses.’’ (P7)

### Information

#### Quality of information provided

A large number of respondents stated that they reach for these applications when they are looking for information, especially respondents who used apps related to medicines or skin care. Users of drug-related apps particularly emphasized the availability of a large volume of up-to-date information and tools relevant to their work or education.

…I like this app because it represents the pharmacotherapeutic guide and the national drug register in one and it is quite easy to search [for information] (P12)

Users of apps related to healthy lifestyles, such as fitness apps, were highlighting that they like having the app track information about their performance or attributes (such as steps, mileage, or weight).

I like the fact that it shows the number of steps [they made], it also shows kilometres, it calculates the average for a week, a month, so it’s interesting for me to keep track [of their performance].” (P21)

However, some respondents felt that certain apps did not provide enough precision in measurement or information about the actual use of the app, such as distance travelled, how to hold the phone while counting steps, or how to use certain tools within medication use apps.

#### Data privacy and data storage in mobile device memory

Most participants explained that they did not consider the app they were using to invade their data privacy, nor that it was a problem for them. Users of apps related to skin care, as well as some other respondents, pointed out that the app had optional identification, so it was not necessary to leave the name and surname if the user did not want to do so. A few respondents pointed out that they don’t like when an app asks for location access or access to information available on their smartphone, but they still accept that because they want to use the app and feel that the benefit outweighs the potential risks.

Every app nowadays asks for access to phone data, I just don’t like it, I try, whenever possible, to instal the application and avoid it, but sometimes the app does not allow to be installed without consent. At that moment, I agree because I need the application. (P16)

The respondents had no problem with the memory that the apps take up.

### Engagement

#### Interactions

Users of different types of apps used the apps during different daily activities. Drug-related app users used such apps mostly during work hours as support to their work or, in case of students, to help with studying. On the other hand, users of fitness apps and running apps used apps during physical activity, while users of apps for monitoring and managing calorie intake and/or weight loss used apps to keep track of their meals. Users of pedometer apps mostly had the app on all day to count the steps they took. When it came to apps related to skin care or pregnancy, respondents used them when in need for information.

When I need [information about] a medicine, I type it in, it [the app] throws out information about all the doses [of the medicine] that exist and it is clearly indicated which medicine is on the list [of prescription drugs], which I like [about the app]. I mostly use this app when I need to find parallels [of drugs]. When I type in the active substance, I look at a series of parallels that I can offer to the patient and dispense at an appropriate price…’’ (P16)

### Motivation and satisfaction

Participants used the apps on their own initiative, either privately or professionally, as those facilitated their access to the required information or the achievement of personal goals, especially as certain apps offer the possibility of personalization. A few respondents mentioned they liked that within certain apps there were challenges where they could compete with other users, and this encouraged them to continue using such apps. Also, the possibility for users to leave their feedback had a positive effect on the respondents’ satisfaction. Notifications related to news, reminders or achieved goals encouraged the use of apps by almost all participants.

The biggest advantage is that [the app] is persistent, because we are not persistent in that sense and then it makes you remember something or learn a new [drug] preparation. … And they [the app] often ask for content ratings and recommendations, so they really follow our interests. I like that the most. (P43)

### Perceived usefulness and value for the user

#### Professional and/or educational value

Respondents who used medication-related apps repeatedly emphasized how useful these apps are in their work, and how they facilitate the provision of high-quality timely advice to patients and adequate pharmaceutical health care. Also, the students pointed out that the apps facilitate their studies at the university as such app represent a source of reliable, available and simply presented information.

This is the closest thing to the old [pharmacy] practice, while the special advantages are the advanced technology and much faster updating. So, in one way, it is a logical continuation of something that was out there before, but in a different form. (P41)

Participants with more work experience also mentioned apps related to skin care and healthy lifestyles as something they use in communication with patients, because in this way they motivate the patients to focus on non-pharmacological measures and disease prevention.

I use it for my own needs, and I also instal it for my patients who come to the pharmacy, if they want to do something about their health. Let’s say we have a blood pressure measurement service [in the pharmacy], so then I explain to them [patients] how important physical activity is with the whole story of medication use. Well, that’s how we connect that story, and then it’s encouraging for them to literally stop by the pharmacy to share how many steps they’ve made, how much more they’ll work to increase it and thus be more and more active day after day, so I’m happy about that. (Q49)

#### Personal value

Apps related to physical activity, nutrition and skin care were mostly used by respondents to achieve personal goals related to their appearance or well-being, to encourage and facilitate their personal development, but also simply to feel good. Numerous participants highlighted that they appreciate how the app keeps track of their results and progress (e.g., activity level on a daily basis, running distance and time, calorie intake, hours of sleep,…) which supports them in setting their personal goals as well as keeping their perseverance.

It [the app] completely satisfied my needs, because at any moment I can check how many kilometres I made per day, how active I was and whether I should change something. There is a possibility to monitor my activity and to work on myself in relation to it. (P26)

Some respondents added that by taking better care of themselves, with such applications, it is easier to encourage others in their environment to do so too.

I like the availability of a wide range of information and the ease of use, because basically, I use it and learn more so that I can help others in the pharmacy, my patients—so they can also work on some way of moving their own organism [being more active]. (P 49)

#### Expansion

Many participants had ideas on how the apps they use could be expanded. Users of skin care apps indicated that they would like to see more personalization, more options to assess skin type, as well as search/sort options and information on where to find recommended skin care products. Participants who used drug-related applications pointed out that they would like more search options and information to be added within the application, such as drug parallels, therapeutic guides, dietary supplements, reference values of laboratory results, drug interactions, clarification of which drugs are currently available on the market. One participant stated that he would like the application to be managed by voice, and another respondent pointed out that they believe that these applications should be more interactive and even more adapted to pharmacists in a way to include more tools that could help pharmacists in their work.

Well, let’s say, I think there should be a little more interaction between the user and the application itself. I think that’s missing. And I think that it is precisely pharmacists who need app adaptations. We get some basic information … I think that tools that would be used by pharmacists could be installed. (P 50)

What would I add, that this application lacks, maybe some [drug] interaction checker? That there is a field where you can enter [names of] two drugs and then it comes down to checking whether that interaction is clinically significant or not. Of course, there is always a pharmacist, who evaluates whether it is okay or not to dispense the medicine. But I think that there should definitely be some checker of interactions … Or even more, the name of a generic and a dietary supplement, that is, herbal drugs or active components of a nutritional supplement. (P 42)

Users of fitness and nutrition related apps indicated that they would like to see more types of physical activity listed (e.g., stair climbing), a greater variety of training levels and plans, more opportunities to personalize content, an easier way to count portions, more audible (musical) stimulation during exercise and better regional/linguistic adaptations.

The only thing that seems bad or difficult for me is that I don’t weigh my food and then I don’t know how much I ate, I take it all in roughly. I would prefer if they could offer me [portion sizes] either in the form of a picture or somehow else to make it simpler. (P 36)

Perhaps a greater variety of training plans [to be offered]. Maybe entering more parameters of my characteristics according to the pace of training that will be recommended to me. (P27)

Several respondents declared that they do not think there is a need to further expand the app possibilities.

I am very satisfied. Especially with the latest version because I remember it from when it was only possible to search for drugs. It is reliable, it has the latest data. I wouldn’t change a thing; I like that some benchmarks were added. I think it gives an overview and perspective to the user. It satisfies all my needs. (P 18)

## Discussion

All mentioned apps were widely and easily available on the market. UX research, with an emphasis on usability, is generally carried out in the initial stages of placing apps on the market to assess how easily the app performs given functions (Interaction Design Forum [homepage on the Internet], 2022). Usability testing is aligned with TAM and HITAM and includes concepts such as efficiency, automation, convenience, entertainment and health literacy suitable for a wide range of consumers (ISO 9241–11, 2018; Anderson et al., 2016). Participants felt that the discussed apps were very fast and easy to use, which is, to some extent, expected as these apps have been on the market for a long time and there was a good chance that their usability has been significantly optimized (Interaction Design Forum [homepage on the Internet], 2022). Pharmacists outlined that the app speed is very important to them as it supports them in providing advice to patients in a time-efficient manner. The participants generally had no problems with health terminology, which was also expected, considering them being pharmacists and pharmacy students, but they mentioned that they have encountered some content-level challenges (complex/foreign language and tools) which they recognized as room for app optimization. Apps being recognized as complicated and unavailable in the local language can be a challenge, especially if these apps were to be recommended to the general population because patients appreciate communication and care in their mother tongue (Hemberg & Sved, 2021) and most users of pharmaceutical services are not digitally literate (International Pharmaceutical Federation [homepage on the Internet], 2022). This is especially important for the Republic of Serbia, considering that in the report from 2019, it was stated that almost one quarter of respondents never used the internet (Team for social inclusion and poverty reduction of the Government of the Republic of Serbia [homepage on the Internet], 2022).

Information management is aligned with HITAM and describes reliability, data privacy, data security, as well as data quality and quantity. mHealth handles sensitive data, and without acceptable information management processes, health apps would not be able to manage data safely and effectively (Anderson et al., 2016; Kim & Park, 2012), therefore privacy and security of users’ data have been a subject of great concern (Nurgalieva et al., 2020). Participants in this study were generally satisfied with the quality, recency and volume of information, while they expressed concerns about data privacy, but mostly concluded that the usefulness of their apps outweighed their potential risks. They explained that they find apps filled with relevant tools and information very practical and that such apps support them in their work, education and in achieving their personal goals by keeping them informed and expanding their knowledge and awareness. Participants from all clusters accentuated perceived usefulness of the apps, especially due to data reliability. This is in line with the findings from study conducted with pharmacists in United Kingdom, which rated the reliability as the most important aspect of the mobile app design (Davies et al., 2015).

MARS and HITAM-compliant mobile app functionality includes app-related instructions, aesthetics, appearance, navigation, and tactile feedback (Anderson et al., 2016). Most of the respondents were satisfied with the consistency and aesthetics of the applications, sharing their partiality towards a simple design with high legibility, as well as visual over tactile feedback. According to MARS, the engagement theme covers interactions between users and applications, motivation to maintain the use, and social factors that enable competition with other users. Apps that can maintain positive behaviour and adapt to changes in consumer demand are more likely to be used on an ongoing basis (Anderson et al., 2016; Stoyanov et al., 2015). Lack of motivation is a commonly mentioned challenge when implementing mHealth into pharmacy care (Chong et al., 2019), however a large number of participants in this study used the apps on a daily basis, as they found motivation and satisfaction in achieving personal goals and/or providing more effective pharmaceutical care, as evidenced by the fact that many of the aforementioned apps have been in use for years. Some added that being able to compete with other users increases their motivation to use the app, while others brought up that apps help them motivate their environment by example i.e., motivate their acquaintances or patients to work on disease prevention, non-pharmacological measures, self-care and their wellbeing in general.

Younger pharmacists are generally more enthusiastic about using health apps in public health (Crilly et al., 2019). However, in this study, the older participants did not fall behind. No significant difference was observed when comparing Cluster III to Clusters I and II, with the fact that for older respondents, their work experience may have even facilitated the use of medication-related apps and enabled easier navigation.

Although generally satisfied, respondents had numerous ideas for expanding the possibilities within the applications and they’ve displayed enthusiasm to participate in app optimization by sharing their experience and insights. Partnerships between pharmacists, researchers and start-up companies or communities could help further development of health apps in line with the themes identified in the research (Anderson et al., 2016).

## Strengths and limitations of the research

The pharmacists involved in this qualitative study were experienced and proactive community pharmacists, and considering pharmacists’ views provides a good starting point for exploring the views of other stakeholders in the future (Nabergoj Makovec et al., 2018). This research also provides insights into the use of mobile applications by pharmacists and pharmacy students from low- and middle-income countries (in this case, Serbia) as the end-users of apps, which is rarely found in the literature. This study supports the importance of UX research as an essential part of introducing smartphone apps into pharmaceutical care (Davies et al., 2015). MARS, TAM and HITAM were used to develop the interview, which provide a broader and more comprehensive insight into the participants’ experience in relation to the use only one model (Anderson et al., 2016).

The enrolled research participants helped in the further recruitment of subjects for the study (snowball sampling), and conducting interviews within a certain network could lead to bias and insufficient distribution of the target population (Parker et al., 2019). In addition, the transcript and quotes from the interviews were not returned to the participants for verification. This could have reduced the level of credibility of the results (Forbat & Henderson, 2005; Nabergoj Makovec et al., 2018). The data analysis was carried out manually, which could also create a chance for bias (Basit, 2003), and significantly extend the coding process. Another limitation of this study was the extended duration of the research, as well as the time gap between conducting the interviews with Clusters I and II and Cluster III which was added later to complete the perspective, which could have caused difficulties in recalling the interviews. This could have affected the reliability of the results and must be taken into account in future studies (Siu et al., 2021).

This study is not able to link user experiences with the credibility of the health apps, and the study was limited to the perspective of pharmacists and pharmacy students from Serbia. Accordingly, apps used internationally are likely to contain different UX metrics. This study did not quantify participants’ experiences, which would be of greater benefit and significance when studying a single application (Anderson et al., 2016). Additionally, another widely used technology acceptance model, the Unified Theory of Acceptance and Use of Technology (UTAUT), which was based on TAM among other models, wasn’t used when developing the interview guide. UTAUT, but even more importantly revised models based on UTAUT who take attitude of individuals into account have a greater ability to explain the factors affecting the users’ adoption of technologies than TAM and using such models could have provided better understanding and insights into the user experience of the participants (Dwivedi et al., 2019).

It is not known whether male and female users of health apps differ in their use of and expectations from these apps. The current sample consisted mainly of female participants, probably due to the recruitment methods, but also because the majority of pharmacists/pharmacy students in Serbia are female. Taking into the account the confirmability criteria in qualitative research we expect the similar results with the same cohort of participants in the same setting. However, transferability of our results within different cohort of healthcare professionals and/or students could be debatable (Morse, 2015).

This research aimed to explore what kind of health apps were used by pharmacists and pharmacy students in Serbia, therefore it was not feasible to focus the study on a single application. Assessing the clinical contribution of healthcare apps requires careful experimental design and control of environmental influence on self-management of the health condition of interest (Anderson et al., 2016).

## Conclusion

The data obtained from this research indicates that experienced and early career pharmacists, as well as pharmacy students use mobile apps related to health, privately, but also as support in education or work, like a source of updated information and tools. During working hours, participants mostly used drug-related apps, which was expected considering that the participants in this research were pharmacists. However, it was also shown that they use other subtypes of applications related to health to provide the best possible care to patients. On the other hand, in their free time, participants used mHealth to work on themselves. This implies the need for further research in this direction, the potential of mobile apps related to health and the necessity of their further development and optimization to provide additional support to pharmacists, both as professionals and individuals, on the path of their continuous improvement. Expanding and optimizing health apps based on feedback obtained in this study could lead to better coverage of users’ i.e., pharmacists’ needs, therefore this study could be iterated when introducing updates or new platforms so there is constant awareness about the room for optimization. An adaptation of the interview guide to include adapted and enhanced technology acceptance research models could provide better insights into user experience. Further exploration of the importance and potential of health apps, especially in the context of pharmacy, should include a focused pilot project research, involving more stakeholders and taking into account the benefits for the patient, but also for the health system and healthcare professionals.

## Data Availability

The data that support the findings of this study are available from the corresponding author upon reasonable request.

## Geolocation information

44.747169773358095, 20.494321876896812
